# Fast food consumption and its associations with obesity and hypertension among children: results from the baseline data of the Childhood Obesity Study in China Mega-cities

**DOI:** 10.1186/s12889-017-4952-x

**Published:** 2017-12-06

**Authors:** Yaling Zhao, Liang Wang, Hong Xue, Huijun Wang, Youfa Wang

**Affiliations:** 1 Global Health Institute; Department of Epidemiology and Biostatistics, School of Public Health, Xi’an Jiaotong University Health Science Center, Xi’an, Shaanxi 710061 China; 2 Department of Biostatistics and Epidemiology, College of Public Health, East Tennessee State University, Johnson City, TN 37614 USA; 3 Department of Health Behavior and Policy, School of Medicine, Virginia Commonwealth University, Richmond, VA 23284 USA; 4 National Institute for Nutrition and Health, Chinese Center for Disease Control and Prevention, Beijing, 100050 China

**Keywords:** Fast food consumption, Obesity, Overweight, Hypertension, Child, Adolescent, China

## Abstract

**Background:**

China has seen rapid increase in obesity and hypertension prevalence and fast food consumption over the past decade. We examined status and risk factors for Western- and Chinese fast food consumption and their associations with health outcomes in Chinese children, and examined how maternal factors were associated with child health outcomes.

**Methods:**

Data of 1626 students aged 7–16 (11.6 ± 2.0) years and their parents in four mega-cities across China (Beijing, Shanghai, Nanjing, and Xi’an) were collected in the 2015 baseline survey of the Childhood Obesity Study in China Mega-cities. Weight, height, waist circumference, and blood pressure were measured. Food intake was assessed using questionnaire. Mixed models were used to examine the associations.

**Results:**

Among the children, 11.1% were obese, 19.7% were centrally obese, and 9.0% had hypertension. Obesity prevalence was much higher in boys than in girls (15.2% vs. 6.9% and 27.4% vs. 11.7%, respectively, both *P* < 0.001). About half (51.9% and 43.6%) of children consumed Western and Chinese fast food, respectively, over the past 3 months. Compared to those with college or above maternal education level, those with elementary school or below maternal education level were 49% more likely to consume Western fast food (odds ratio [OR] and 95% confidence interval [CI]: 1.49 [1.10–2.03]). Chinese fast food consumption rate increased by 12% with each year of increase in child’s age (OR and 95% CI: 1.12 [1.02–1.23]). No significant associations between fast food consumption and health outcomes were detected. Adjusting for Western fast food consumption, children with lower maternal education were 71% and 43% more likely to have obesity and central obesity (ORs and 95% CIs: 1.71 [1.12–2.61] and 1.43 [1.00–2.03], respectively), and maternal body mass index was positively associated with child obesity, central obesity, and hypertension (ORs and 95% CIs: 1.11 [1.06–1.17], 1.12 [1.07–1.17], and 1.09 [1.03–1.15], respectively). Results were similar when Chinese fast food consumption was adjusted for.

**Conclusions:**

The prevalence of fast food consumption, obesity and hypertension is high among children in major cities in China. Maternal factors affect child outcomes.

## Background

The prevalence of obesity and overweight has increased rapidly among Chinese adults and children over the past three decades [1–5]. In children, it increased from less than 3% in 1985 to about 20% in 2010 [4]. It has reached 42.6% in Chinese adults by 2010 [6]. It is much more prevalent in major cities, reaching about 50% in Beijing children in recent years. There are also indicators of worsening of other health problems like elevated blood pressure (BP) [7]. In a recent study based on data collected during 2004–2014, we reported adverse trends in ideal cardiovascular health indicators among Chinese children and adolescents [7]. The ideal levels of almost all the seven metrics, including smoking, diet, body mass index (BMI), and BP, suggested by the American Heart Association’s 2020 Strategic Goals, had decreased among Chinese Children [7]. According to a recent report, 20.2% of Chinese boys and 16.3% of girls had elevated BP; elevated BP was also common among obese children [8]. This is much higher than among American children [9]. The shifts in Chinese children’s food intake might have contributed to the increase in obesity and hypertension.

The fast food (FF) industry and people’s fast food consumption (FFC) have grown rapidly in China [10]. The number of McDonald’s alone rose from 1 to 1000 between 1990 and 2006 [10, 11]. At present, ‘Yum! China’ has approximately 4800 KFCs and 1300 Pizza Huts, with a plan to open around 20,000 restaurants in China [10]. FF is high in unhealthy fats, salt, and sugar, which contributes to obesity and elevated BP [11] and has been positively associated with the obesity epidemic due to its increasing availability, energy density, and large portions [12–14]. Some studies have reported that sodium intake was associated with elevated systolic blood pressure (SBP) and diastolic blood pressure (DBP) both among adults [15–20] and children [21–23].

In Western countries, although a positive association has been suggested between frequent FFC and weight gain in adults [24–26], there is limited and mixed evidence for children [27–32]. Some studies have shown a small association between FFC and increased BMI [12, 27–29], while others did not detect a significant association [30–32]. One multicenter, multi-country cross-sectional study of 199,135 adolescents aged 13–14 years, including adolescents from Mainland China and Taiwan, and 72,900 children aged 6–7 years, including children from Taiwan but without children from Mainland China, suggested that children’s BMI was higher for those who consumed FF more frequently, however, adolescents’ BMI was lower for those who consumed FF more frequently [13]. Two systematic reviews suggest the need for more studies using larger study samples and adjusting for confounding factors such as parental BMI [12, 13].

Some previous studies have examined the association between FFC and obesity in China, while most previous research has focused on Western FFC. Some reported a positive association [10, 33–37], while some did not [38, 39]. They are predominately cross-sectional studies and are based on local samples. Few have studied the predictors of FFC and the association between FFC and other health outcomes such as elevated BP.

To address these research gaps, this study aimed to: a) examine the current FFC (both Western FF and Chinese FF) patterns among school-age children in four mega-cities across China; b) explore factors being associated with FFC; and c) assess the association between FFC and selected health outcomes (i.e., obesity, central obesity, and elevated BP). We also examined gender differences in FFC and health outcomes among children, and how maternal factors may be associated with children’s FFC and having obesity and elevated BP.

## Methods

### Study design and participants

The Childhood Obesity Study in China Mega-cities is a longitudinal study aimed at examining the etiology of childhood obesity and chronic diseases in China, especially in its major cities, which have been experiencing rapid economic and social transitions over the past three decades. These transitions have resulted in many dramatic changes in the social environments and in people’s lifestyles, which have led to the increase in obesity and other health problems, such as elevated BP.

Initially, four major cities across China were selected in 2015 for the study, including Beijing (China’s capital, in North China), Shanghai (the largest city in China, in Southeast China), Nanjing (China’s old capital before 1949, in Southeast China), and Xi’an (Northwest China, which has served as the capital of China for 13 dynasties and over 1300 years).

The 2015 October baseline data were collected from 1648 students plus their parents and related school administrators, physical education teachers, and/or school nurses from 16 schools (8 primary schools and 8 middle schools). Four schools (two primary schools and two middle schools) were selected at random in each city. In each selected primary school, one class was selected at random from the 3rd to the 6th grades, while in each middle school, one class was selected at random from the 7th to the 9th grades. Data were collected through questionnaires and direct measurements (i.e., students’ anthropometric measures and BP). The data contain rich information such as child growth and health, family characteristics, home, community and school environment, dietary intake, physical activity, and social networks. The present analysis focused on 1626 students with key variables, such as students’ age, sex, FFC, height, weight, BP, and maternal education level.

The study was approved by the Ethical Committee of The State University of New York at Buffalo (FWA00008824) and related collaboration institutes in China. Consent from parents and school administrators and assent from children were obtained before investigation.

### Key study variables

#### Health outcome variables

Obesity, central obesity, and elevated BP were key outcome variables. Anthropometric measures and BP data were obtained through a physical examination conducted specifically for this study by trained personnel (physicians and nurses) using standardized protocols and calibrated equipment. Height was measured by a Seca 213 Portable Stadiometer Height-Rod with a precision of 0.1 cm; body weight was measured by a Seca 877 electronic flat scale with a precision of 0.1 kg; and waist circumference was measured by a tape with a precision of 0.1 cm.

BMI was calculated as weight in kilograms divided by height in meters squared. Obesity and overweight were defined using the Working Group on Obesity in China (WGOC) age-sex**-**specific BMI cutoffs, which were developed based on age-sex-specific BMI curves that correspond to BMI cutoffs of 24 and 28 at age 18 [40]. BMI cutoffs of 24 and 28 were used to define overweight and obesity, respectively, for adults in China. Waist-height ratio (WHtR) ≥ 0.48 was used to define central obesity for Chinese children [41].

BP was measured twice with an Omron HBP-1300 professional BP monitor. The second of the two measures was recorded if the difference between the two was less than 10 mmHg. A third measure was performed if the difference between the first two measures was more than 10 mmHg, and any two measures with a difference less than 10 mmHg were recorded. Based on Chinese national data, BP references (sex-age-specific SBP and DBP percentiles) for Chinese children and adolescents have been established [42]. They were applied in this study to define elevated BP (otherwise called hypertension) as SBP and/or DBP ≥ 95th percentiles.

#### Fast food consumption

We assessed Western and Chinese FFC. Western FFC was determined by responding to the question, “How often (times/week) did you eat a meal or snack in Western-style FF restaurants (e.g., McDonald’s, KFC, Pizza Hut) in the past three months?” Chinese FFC was determined by answers to the question, “How often (times/week) did you eat a meal or snack in a food stall or non-Western FF restaurant in the past three months?” Each FFC pattern was categorized as yes/no, and times of FFC per week (0, 1–2, and ≥ 3 times).

#### Other covariates

Student factors included school level (primary school, middle school), sex, age, and location (Beijing, Shanghai, Nanjing, and Xi’an). Maternal factors included BMI and education level. Maternal BMI was obtained from self-reported height and weight. Maternal education levels included elementary school or below, middle, high or vocational schools, and college or above.

### Statistical analysis

First, characteristics of participants were described, for continuous variables, presented as means (standard deviation, SD); for categorical variables, as counts and percentages. Chi-square and t-tests were used to determine whether there was a difference by characteristics such as child’s sex and type of schools. Second, descriptive analysis was used to provide an overview of the current FFC pattern (i.e., Western and Chinese) and health outcomes (i.e., obesity, central obesity, and hypertension) in Chinese children by school, gender, and city.

Finally, mixed-effects models were fitted to examine risk factors of FFC patterns as well as the association between FFC and health outcomes, adjusting for child factors (age, sex, and school location) and maternal factors (BMI and education level). The initial models included FFC or health outcomes as the dependent variables. Then, school was included in the models as a random effect (intercept). Including school as a random effect in the model resulted in a smaller Bayesian information criterion, indicating improved model fit.

All analyses were performed using SAS Version 9.4 (SAS Institute, Cary, NC, USA). A significance level of *P* < 0.05 was used.

## Results

### Study participants’ characteristics

The child’s socio-demographic characteristics (age, location, and school level) and health outcome variables and mothers’ BMI and education levels are shown in Table 1. The 1626 investigated students were 7–16 (11.6 ± 2.0) years old. About half (51.0%) of the students were boys. About half (51.6%) of them were primary school students, and 48.4% were middle schoolers.

**Table 1:** Characteristics of Chinese children aged 7–16 years in the study

Characteristics	Overall (*n* = 1626)	Boys (*n* = 830)	Girls (*n* = 796)	*P*-value
Child health outcomes				
Obesity related variables				
BMI, mean (SD)	19.2 (3.9)	19.7 (4.0)	18.6 (3.8)	< 0.001
Waist circumference, mean (SD)	66.0 (10.3)	68.2 (11.1)	63.7 (9.0)	< 0.001
WHtR, mean (SD)	0.44 (0.06)	0.45 (0.06)	0.42 (0.05)	< 0.001
Blood pressure				
Systolic blood pressure, mean (SD)	106.0 (12.1)	107.2 (12.4)	104.8 (11.6)	< 0.001
Diastolic blood pressure, mean (SD)	60.9 (7.9)	60.4 (7.7)	61.4 (8.1)	0.017
Child characteristics				
Age (in years), mean (SD)	11.6 (2.0)	11.6 (2.0)	11.5 (2.1)	0.917
Location, n (%)				0.618
Beijing	439 (27.0)	213 (25.7)	226 (28.4)	
Shanghai	400 (24.6)	212 (25.5)	188 (23.6)	
Nanjing	396 (24.4)	204 (24.6)	192 (24.1)	
Xi’an	391 (24.1)	201 (24.2)	190 (23.9)	
School level, n (%)				0.745
Primary school	839 (51.6)	425 (51.2)	414 (52.0)	
Middle school	787 (48.4)	405 (48.8)	382 (48.0)	
Their mothers’ characteristics				
BMI, mean (SD)	22.1 (3.2)	21.9 (2.8)	22.4 (3.4)	0.001
Education level, n (%)				< 0.001
Elementary school or below	402 (25.6)	242 (30.5)	160 (20.7)	
Middle, high or vocational school	452 (28.8)	215 (27.1)	237 (30.6)	
College or above	715 (45.6)	337 (42.4)	378 (48.8)	

Abbreviation: *BMI* body mass index, *SD* standard deviation, *WHtR* waist-height ratio

### Prevalence of fast food consumption and health outcomes

Table 2 shows the patterns of FFC, which was common, i.e., 51.9% reported having Western FFC at least once per week over the past 3 months. There was no difference observed between primary school students and middle school students (*P* = 0.756), between boys and girls (*P* = 0.656), or among students from different cities (*P* = 0.464). 43.6% of students had Chinese FFC at least once per week. Fewer primary school students consumed Chinese FF frequently compared to middle school students (35.3% consumed 1–2 times per week and 2.8% consumed ≥ 3 times per week vs. 44.4% and 4.4%, respectively, *P* < 0.001), while more boys consumed Chinese FF frequently than girls (41.6% consumed 1–2 times per week and 4.5% consumed ≥3 times per week vs. 38.5% and 2.7%, respectively, *P* = 0.044).

**Table 2:** Prevalence of fast food consumption and health outcomes among children in the study (*n* = 1626)^ab^

Characteristics	Had FFC each week (%)		Times of FFC per week (%)						Health outcomes, yes (%)			
	Western FFC	Chinese FFC	Western FFC			Chinese FFC			Overweight (including obesity)	Obesity	Central obesity	Hypertension
			Not consumed	1–2 time	≥ 3 times	Not consumed	1–2 times	≥ 3 times				
All	51.9	43.6	48.1	36.3	15.6	56.4	40.0	3.6	26.2	11.1	19.7	9.0
School												
Primary school	51.5	**38.1**	**48.5**	**39.2**	**12.3**	**61.9**	**35.3**	**2.8**	26.9	12.3	20.1	5.7
Middle school	52.2	**48.8**	**47.8**	**33.7**	**18.5**	**51.2**	**44.4**	**4.4**	25.5	9.9	19.2	12.7
*P*-value	0.756	**< 0.001**	**0.001**			**< 0.001**			0.529	0.134	0.625	< 0.001
Gender												
Boys	51.3	46.2	48.8	36.8	14.5	**53.9**	**41.6**	**4.5**	**33.9**	**15.2**	**27.4**	9.3
Girls	52.4	41.3	47.6	35.8	16.6	**58.8**	**38.5**	**2.7**	**18.2**	**6.9**	**11.7**	8.8
*P*-value	0.656	0.051	0.507			**0.044**			**< 0.001**	**< 0.001**	**< 0.001**	0.734
City												
Beijing	49.8	44.5	50.2	31.7	18.1	**55.5**	**40.3**	**4.2**	28.9	11.9	19.1	9.1
Shanghai	51.9	45.0	48.2	36.1	15.7	**55.6**	**41.1**	**3.3**	27.3	10.5	23.0	7.5
Nanjing	55.1	46.1	44.9	38.5	16.6	**53.9**	**40.5**	**5.6**	21.7	10.6	20.7	11.9
Xi’an	50.8	39.2	49.2	39.7	11.2	**60.8**	**38.1**	**1.1**	26.6	11.5	15.9	7.7
*P*-value	0.464	0.235	0.059			**0.039**			0.108	0.907	0.080	0.117

Abbreviations: *FFC* fast food consumption

^a^Column % was used for comparison

^b^The differences across groups were tested using Chi-square or t-tests. Those bolded were *P* < 0.05 for the group differences

The prevalence of overweight (including obesity), obesity, and central obesity of the study students was 26.2%, 11.1%, and 19.7%, respectively. The rates for boys were higher than those for girls (33.9%, 15.2%, and 27.4%, vs. 18.2%, 6.9%, and 11.7%, respectively, all *P* < 0.001). An elevated BP rate was found in 9.0% of the students, with no significant difference by gender or among the cities (*P* = 0.734 and 0.117, respectively).

### Factors associated with fast food consumption

Mixed model analysis (see Table 3) shows that low maternal education was positively associated with Western FFC. Children having mothers with an elementary school or below education level were more likely to consume Western FF than those with college or above maternal education (odds ratio [OR] and 95% confidence interval [CI]: 1.49 [1.10–2.03]). Child’s age, gender, city living, and maternal BMI were not significantly associated with Western FFC. As age increased, children were more likely to consume Chinese FF (OR and 95% CI: 1.12 [1.02–1.23]).

**Table 3:** Factors associated with Western and Chinese fast food consumption among Chinese children (*n* = 1626)^a^

	Western FFC each week (yes vs. no) OR (95% CI)	Chinese FFC each week (yes vs. no) OR (95% CI)
Child factors		
Age	1.07 (0.99–1.14)	1.12 (1.02–1.23)**
Boy (vs. girl)	0.94 (0.75–1.18)	1.18 (0.94–1.49)
City (vs. Beijing)		
Shanghai	0.95 (0.56–1.60)	1.01 (0.35–2.93)
Nanjing	1.08 (0.64–1.84)	0.87 (0.30–2.52)
Xi’an	0.93 (0.54–1.60)	0.71 (0.24–2.07)
Maternal factors		
BMI	1.00 (0.97–1.03)	0.96 (0.93–1.00)*
Education level (vs. college or above)		
Elementary school or below	1.49 (1.10–2.03)*	1.10 (0.79–1.53)
Middle, high or vocational school	1.22 (0.92–1.60)	1.18 (0.89–1.56)

Abbreviations: *FFC* fast food consumption, *OR* odds ratio, *95% CI* 95% confidence interval

^a^Based on mixed models; bold indicates *P* < 0.05; *P < 0.05, **P < 0.01

### Association between fast food consumption and health outcomes

Table 4 shows the prevalence of health outcomes (overweight, obesity, central obesity, and hypertension) by FFC. The associations between FFC and health outcomes were further assessed using mixed models, controlling for some child and maternal factors (see Table 5). No statistically significant association was detected between Western FFC and obesity or central obesity and hypertension, respectively. The results for Chinese FFC were similar to those for Western FFC.

**Table 4:** Prevalence of health outcomes by fast food consumption among Chinese children (*n* = 1626)^a^

Health outcomes	Overweight (Including Obesity, %)			Obesity (%)			Central obesity (%)			Hypertension (%)		
FFC per week	All	Boy	Girl	All	Boy	Girl	All	Boy	Girl	All	Boy	Girl
1) Western FFC												
Ever consumed												
No	27.6	35.6	19.1	11.7	16.2	7.0	20.5	29.0	11.6	8.9	10.3	7.5
Yes	24.9	32.6	17.1	10.7	14.6	6.8	19.1	26.5	11.7	9.3	8.5	10.0
*P*-value	0.220	0.365	0.465	0.544	0.542	0.929	0.501	0.438	0.949	0.818	0.398	0.223
Times												
0	27.6	35.6	19.1	11.7	16.2	7.0	20.5	29.0	11.6	8.9	10.3	**7.5**
1–2	26.0	31.6	20.0	10.6	13.3	7.9	18.6	24.5	12.5	10.5	8.8	**12.1**
≥ 3	22.0	34.5	10.8	11.0	18.1	4.6	19.9	31.0	10.0	6.5	7.8	**5.4**
*P*-value	0.219	0.545	0.059	0.828	0.396	0.482	0.705	0.290	0.762	0.192	0.667	**0.038**
2) Chinese FFC												
Ever consumed												
No	27.5	34.3	**21.1**	10.8	15.1	6.7	19.6	26.2	13.5	**10.6**	10.7	10.4
Yes	24.8	34.1	**14.2**	11.8	15.7	7.4	20.2	29.5	9.6	**7.1**	7.6	6.5
*P*-value	0.224	0.933	**0.014**	0.515	0.816	0.718	0.792	0.307	0.095	**0.016**	0.130	0.054
Times												
0	27.5	34.3	**21.1**	10.8	15.1	6.7	19.6	26.2	13.5	10.6	10.7	10.4
1–2	24.3	33.4	**14.3**	11.5	15.4	7.3	19.6	28.9	9.3	7.1	7.2	7.0
≥ 3	26.3	36.1	**9.5**	15.8	19.4	9.5	22.8	30.6	9.5	7.0	11.1	0.0
*P*-value	0.382	0.932	**0.034**	0.489	0.784	0.863	0.838	0.652	0.206	0.060	0.247	0.091

Abbreviations: *FFC* fast food consumption

^a^Column % was used for comparison; those bolded, *P* < 0.05

**Table 5:** Associations between fast food consumption and health outcomes among Chinese children (*n* = 1626)^a^

	Obesity	Central obesity	Hypertension
	OR (95% CI)	OR (95% CI)	OR (95% CI)
1) Western FFC			
Times of FFC per week (vs. 0)			
1–2	0.96 (0.65–1.41)	0.92 (0.68–1.26)	1.17 (0.78–1.75)
≥ 3	0.89 (0.53–1.49)	0.99 (0.66–1.49)	0.68 (0.35–1.31)
Child factors			
Age	0.92 (0.84–1.00)	0.95 (0.88–1.03)	1.33 (1.15–1.52)***
Boy (vs. girl)	2.63 (1.79–3.89) ***	3.15 (2.31–4.31)***	1.21 (0.81–1.81)
City (vs. Beijing)			
Shanghai	0.95 (0.57–1.60)	1.29 (0.78–2.12)	0.81 (0.29–2.27)
Nanjing	0.75 (0.43–1.29)	0.97 (0.58–1.62)	1.73 (0.64–4.66)
Xi’an	0.95 (0.55–1.64)	0.78 (0.45–1.34)	0.89 (0.32–2.49)
Maternal factors			
BMI	1.11 (1.06–1.17)***	1.12 (1.07–1.17)***	1.09 (1.03–1.15)**
Education level (vs. College or above)			
Elementary school or below	1.05 (0.66–1.67)	1.05 (0.71–1.53)	0.81 (0.46–1.44)
Middle, high or vocational school	1.71 (1.12–2.61)*	1.43 (1.00–2.03)*	1.34 (0.83–2.15)
2) Chinese FFC			
Times of FFC per week (vs. 0)			
1–2	0.97 (0.68–1.40)	0.94 (0.70–1.26)	0.67 (0.44–1.02)
≥ 3	1.21 (0.50–2.95)	0.97 (0.46–2.07)	0.92 (0.30–2.84)
Child factors			
Age	0.92 (0.84–1.00)	0.96 (0.88–1.03)	1.34 (1.17–1.54)***
Boy (vs. girl)	2.64 (1.79–3.89)***	3.16 (2.31–4.32)***	1.24 (0.83–1.85)
City (vs. Beijing)			
Shanghai	0.96 (0.57–1.61)	1.29 (0.78–2.13)	0.81 (0.28–2.32)
Nanjing	0.75 (0.43–1.29)	0.96 (0.57–1.62)	1.71 (0.62–4.71)
Xi’an	0.96 (0.56–1.66)	0.77 (0.45–1.34)	0.89 (0.31–2.55)
Maternal factors			
BMI	1.11 (1.06–1.17)***	1.12 (1.07–1.17)***	1.09 (1.03–1.14)**
Education level (vs. College or above)			
Elementary school or below	1.03 (0.65–1.65)	1.04 (0.71–1.52)	0.80 (0.45–1.43)
Middle, high or vocational school	1.70 (1.11–2.60)*	1.43 (1.00–2.03)*	1.33 (0.83–2.14)

Abbreviations: *FFC* fast food consumption, *OR* odds ratio, *95% CI* 95% confidence interval

^a^Based on mixed models; bold indicates *P* < 0.05; *P < 0.05, **P < 0.01, ***P < 0.0001

Some child and maternal factors were associated with these health outcomes. Adjusting for Western FFC, boys had a higher likelihood of being obese (OR and 95% CI: 2.63 [1.79–3.89]) and centrally obese (OR and 95% CI: 3.15 [2.31–4.31]) than girls. With an increase in maternal BMI, children were more likely to be obese (OR and 95% CI: 1.11 [1.06–1.17]) and have central obesity (OR and 95% CI: 1.12 [1.07–1.17]). Compared with children whose mothers had > = college education, those with lower maternal education were more likely to be obese (OR and 95% CI: 1.71 [1.12–2.61]) and centrally obese (OR and 95% CI: 1.43 [1.00–2.03]). With an increase in child age (OR and 95% CI: 1.33 [1.15–1.52]) and in maternal BMI (OR and 95% CI: 1.09 [1.03–1.15]), children were more likely to have an elevated BP. Results were similar when Chinese FFC was adjusted for.

## Discussion

Using data collected from four mega-cities across China, we have reported on FFC and its associated factors among elementary and middle school students. This is one of the first studies to examine the associations between FFC and obesity, central obesity, and hypertension among students in mega-cities across China. FFC is prevalent among children in mega-cities, and has been increasing steadily over the past decade. Chinese children in urban areas consumed FF much more frequently than those in rural areas. About 50% of our studied students reported having consumed Western FF (51.9%) and/or Chinese FF (43.6%) at least once per week over the past 3 months. The 9-province China Health and Nutrition Survey data reported that 18.5% (38.0% in urban vs. 11.0% in rural areas) of school children had consumed Western FF over the past 3 months in 2004 and 23.9% (43.3% in urban vs. 15.9% in rural) in 2009 [39].

Our study found that 11.1% of these students were obese, while 26.2% were overweight or obese. The prevalence was higher than the national average. Based on the 2010 Chinese National Surveys on Students’ Constitution and Health data, the national overweight/obesity prevalence was 22.6% in urban areas and 16.2% in rural areas [4].

The hypertension prevalence was 9.0% in these Chinese students, which is similar to that in American children but lower than Chinese national prevalence. According to a recent report, 20.2% of Chinese boys and 16.3% of girls had elevated BP [8]. The China Health and Nutrition Survey data reported that hypertension prevalence among children in 2009 was 16.2% in urban areas and 13.0% in rural areas [43]. Differences in BP measurements and the study populations could have contributed to these differences. The U.S. National Health and Nutrition Examination Survey data showed that mean BP and prevalence of elevated BP among U.S. children and adolescents had declined during 1999–2012, although the prevalence of obesity/overweight had increased. In 2009–2012, the prevalence of elevated BP and hypertension in U.S. children and adolescents were 9.6% and 1.6%, reduced by 2.8% and 1.3% from 1999 to 2002, respectively [9]. These declines among U.S. children and adolescents might be associated with decrease in some nutritional factors (e.g., daily intakes of energy, carbohydrate, total fat, and total saturated fatty acids), and increase in daily intake of total polyunsaturated fatty acids and dietary fiber [9]. However, Xi et al. reported that the mean BP and the prevalence of pre-hypertension and hypertension among Chinese children age 6–17 years increased significantly from 1993 to 2009, and the increases could be partially attributed to the increases in general and central obesity, salt intake and sedentary behavior, and decrease in physical activity [43]. More studies are warranted to examine the trend and risk factors for hypertension among children in China.

In China, over the past two decades, rapid economic development, global trade, and cultural exchange have meant that the FF industry and children’s FFC have been increasing rapidly [10]. However, we did not detect associations between FFC and obesity, central obesity, and hypertension. Inconsistent with results of the multicenter, multi-country cross-sectional study which showed FFC was positively associated with children’s BMI but negatively associated with adolescents’ BMI [13], our results found neither Western nor Chinese FFC was associated with child BMI (data were not shown). These may be due to the cross-sectional data, which cannot assess causality. Overweight children might have changed their dietary patterns, e.g., reduced FFC. This may distort or mask the associations between FFC and obesity.

Previously mixed results based on cross-sectional data have been reported on the association between FFC and obesity among Chinese children. A study among children in Beijing reported that children who consumed FF ≥ 3 times per week were 50% more likely to be obese (OR and 95% CI: 1.50 [1.12–2.02]) compared to those had indulged < 1 time per week [33]. Another study among middle and high school students from seven large cities in China reported a reverse association between Western FFC and overweight-adolescents who were overweight were more likely to consume FF less frequently (OR and 95% CI: 0.93 [0.87–0.99]) [44].

Other reasons for the mixed results being reported on the association between FFC and obesity may include that some studies only examined frequency of FFC and did not consider the quantity of FFC. The contribution of FFC to total energy intake varied considerably between populations. FFC played a much more dominant role in American children’s diet than for children’s diet in China. One study reported that FFC contributed to about 20% of American children’s total energy intake, but only < 0.1% for Chinese children [45]. Nevertheless, a growing body of research suggests FFC contributed to increased caloric intake and obesity risk [10, 12].

Of interest, we also found significant associations between maternal education and BMI and their children’s health outcomes. Higher maternal education and lower maternal BMI seem to be protective. Compared to children with mothers having college or above education level, those whose mothers had elementary school or below education were 49% more likely to consume Western FF. Children of mothers with lower education were 71% and 43% more likely to have obesity and central obesity, respectively. As mothers’ BMI increased, their children were more likely to have obesity, central obesity, and hypertension. This indicates mothers should be a key part of future childhood obesity prevention programs in China.

In this study, we defined Western FF as food sold in these fast food chains, e.g. KFC, McDonald’s, Pizza Hut, and defined food from non-westernized style quick service vendors, including food stalls, as Chinese FF. There may be some non-fried food (e.g., steamed buns and noodle soup) sold in food stalls. This would make our estimate more conservative. However, the components, nutrients, and health consequences of Western and Chinese FF may be different. More studies are needed to identify the differences between Western and Chinese FFC and their single and co-association with obesity, hypertension, and other health-related outcomes.

Our study has several main strengths. First, key outcome variables (i.e., height, weight, waist circumference, and BP) were objectively measured rather than self-reported. Second, some important confounders were controlled for in our models testing the associations between FFC and health outcomes, including maternal BMI and maternal education. Some limitations should be considered when interpreting our results. First, the definition of FFC was determined by eating in Western-style FF restaurants or eating a meal or snack in a food stall or in a non-westernized FF restaurant. In this case, FF bought from supermarket and ate at home may have not been considered. Second, we used modified food frequency questionnaires (FFQ) to estimate FFC instead of using 24 dietary recalls. There were both pros and cons of using these methods. Given the large sample in our study, we chose to use FFQ. So, we could not obtain information on the quantity of FFC, total daily energy intake, and FFC’s contribution to total daily energy intake among the children. It may affect the assessment of the relationship between FFC and health outcomes. Third, we could not make causal inference due to cross-sectional data structure. Fourth, study participants were 7–16 years old students from four mega-cities across China, which are more developed than other small cities and rural areas of China. Thus, our results could not represent children and adolescents living in small cities or rural areas of China.

Therefore, more studies, especially longitudinal studies based on large national representative samples with exact measures of quantity of FFC intake and its contribution to total daily energy intake, are needed to detect the association between FFC and health outcomes. We are collecting follow-up data which would allow us to conduct longitudinal studies in the future.

## Conclusions

The prevalence of FFC, obesity, and hypertension is high among children in mega-cities in China. The obesity prevalence was much higher in boys, and the FFC and hypertension prevalence was higher in older children. Maternal factors affect child FFC and health outcomes. Children of mothers with low education were more likely to have FFC, and with the increase in maternal BMI, children were more likely to be obese and have hypertension. Associations between FFC and obesity, central obesity, and hypertension were not detected. More studies are warranted. To fight the epidemic of obesity, hypertension, and increasing FFC among children, national and regional programs and policies are needed to create a healthy food environment and promote the development of healthy lifestyles in young people. School-based programs are also needed to provide effective health education and health promotion for children. More attention should be given to boys, and health education should begin at young ages and cover parents.

## Abbreviations

BMI: Body mass index
BP: Blood pressure
CI: Confidence interval
DBP: Diastolic blood pressure
FF: Fast food
FFC: Fast food consumption
FFQ: Food frequency questionnaires
OR: Odds ratio
SBP: Systolic blood pressure
SD: standard deviation
WHtR: waist-height ratio

## References

[1] Wang Y, Lobstein T (2006). Worldwide trends in childhood overweight and obesity. Int J Pediatr Obes.

[2] Wang Y, Mi J, Shan XY, Wang QJ, Ge KY (2007). Is China facing an obesity epidemic and the consequences? The trends in obesity and chronic disease in China. Int J Obes.

[3] Wang Y, Wang L, Qu W (2017). New national data show alarming increase in obesity and noncommunicable chronic diseases in China. Eur J Clin Nutr.

[4] Sun H, Ma Y, Han D, Pan CW, Xu Y (2014). Prevalence and trends in obesity among China's children and adolescents, 1985-2010. PLoS One.

[5] Mi YJ, Zhang B, Wang HJ, Yan J, Han W, Zhao J (2015). Prevalence and secular trends in obesity among Chinese adults, 1991-2011. Am J Prev Med.

[6] Li XY, Jiang Y, Hu N, Li YC, Zhang M, Huang ZJ (2012). Prevalence and characteristic of overweight and obesity among adults in China, 2010. Zhonghua Yu Fang Yi Xue Za Zhi.

[7] Dong H, Yan Y, Liu J, Zhao X, Cheng H, Hou D (2017). Alarming trends in ideal cardiovascular health among children and adolescents in Beijing, China, 2004 to 2014. Int J Cardiol.

[8] Zhai Y, Li WR, Shen C, Qian F, Shi XM (2015). Prevalence and correlates of elevated blood pessure in Chinese children aged 6-13 years: a nationwide school-based survey. Biomed Environ Sci.

[9] Xi B, Zhang T, Zhang M, Liu F, Zong X, Zhao M (2016). Trends in elevated blood pressure among US children and adolescents: 1999-2012. Am J Hypertens.

[10] Wang Y, Wang L, Xue H, Qu W. A review of the growth of the fast food industry in China and its potential impact on obesity. Int J Environ Res Public Health. 2016;13(11):E1112. doi:10.3390/ijerph13111112. 10.3390/ijerph13111112 PMC5129322 27834887

[11] Pan A, Malik VS, Hu FB (2012). Exporting diabetes mellitus to Asia: the impact of western-style fast food. Circulation.

[12] Rosenheck R (2008). Fast food consumption and increased caloric intake: a systematic review of a trajectory towards weight gain and obesity risk. Obes Rev.

[13] Braithwaite I, Stewart AW, Hancox RJ, Beasley R, Murphy R, Mitchell EA (2014). Fast-food consumption and body mass index in children and adolescents: an international cross-sectional study. BMJ Open.

[14] Poti JM, Duffey KJ, Popkin BM (2014). The association of fast food consumption with poor dietary outcomes and obesity among children: is it the fast food or the remainder of the diet?. Am J Clin Nutr.

[15] De Santo NG (2014). Reduction of sodium intake is a prerequisite for preventing and curing high blood pressure in hypertensive patients - second part: guidelines. Curr Hypertens Rev.

[16] Chateau-Degat ML, Ferland A, Dery S, Dewailly E (2012). Dietary sodium intake deleteriously affects blood pressure in a normotensive population. Eur J Clin Nutr.

[17] Zhang Z, Cogswell ME, Gillespie C, Fang J, Loustalot F, Dai S (2013). Association between usual sodium and potassium intake and blood pressure and hypertension among U.S. adults: NHANES 2005-2010. PLoS One.

[18] Noh HM, Park SY, Lee HS, Oh HY, Paek YJ, Song HJ (2015). Association between high blood pressure and intakes of sodium and potassium among Korean adults: Korean National Health and nutrition examination survey, 2007-2012. J Acad Nutr Diet.

[19] Ravi S, Bermudez OI, Harivanzan V, Kenneth Chui KH, Vasudevan P, Must A (2016). Sodium intake, blood pressure, and dietary sources of sodium in an adult south Indian population. Ann Glob Health.

[20] Mohammadifard N, Khaledifar A, Khosravi A, Nouri F, Pourmoghadas A, Feizi A (2017). Dietary sodium and potassium intake and their association with blood pressure in a non-hypertensive Iranian adult population: Isfahan salt study. Nutr Diet.

[21] Lava SA, Bianchetti MG, Simonetti GD (2015). Salt intake in children and its consequences on blood pressure. Pediatr Nephrol.

[22] Farajian P, Panagiotakos DB, Risvas G, Micha R, Tsioufis C, Zampelas A (2015). Dietary and lifestyle patterns in relation to high blood pressure in children: the GRECO study. J Hypertens.

[23] Appel LJ, Lichtenstein AH, Callahan EA, Sinaiko A, Van Horn L, Whitsel L (2015). Reducing sodium intake in children: a public health investment. J Clin Hypertens (Greenwich).

[24] Duffey KJ, Gordon-Larsen P, Jacobs DR, Williams OD, Popkin BM (2007). Differential associations of fast food and restaurant food consumption with 3-y change in body mass index: the coronary artery risk development in young adults study. Am J Clin Nutr.

[25] Duffey KJ, Gordon-Larsen P, Steffen LM, Jacobs DR, Popkin BM (2009). Regular consumption from fast food establishments relative to other restaurants is differentially associated with metabolic outcomes in young adults. J Nutr.

[26] Bezerra IN, Curioni C, Sichieri R (2012). Association between eating out of home and body weight. Nutr Rev.

[27] Thompson OM, Ballew C, Resnicow K, Must A, Bandini LG, Cyr H (2004). Food purchased away from home as a predictor of change in BMI z-score among girls. Int J Obes Relat Metab Disord.

[28] Niemeier HM, Raynor HA, Lloyd-Richardson EE, Rogers ML, Wing RR (2006). Fast food consumption and breakfast skipping: predictors of weight gain from adolescence to adulthood in a nationally representative sample. J Adolesc Health.

[29] Fraser LK, Clarke GP, Cade JE, Edwards KL (2012). Fast food and obesity: a spatial analysis in a large United Kingdom population of children aged 13-15. Am J Prev Med.

[30] French SA, Story M, Neumark-Sztainer D, Fulkerson JA, Hannan P (2001). Fast food restaurant use among adolescents: associations with nutrient intake, food choices and behavioral and psychosocial variables. Int J Obes Relat Metab Disord.

[31] Boutelle KN, Fulkerson JA, Neumark-Sztainer D, Story M, French SA (2007). Fast food for family meals: relationships with parent and adolescent food intake, home food availability and weight status. Public Health Nutr.

[32] Duncan JS, Schofield G, Duncan EK, Rush EC (2008). Risk factors for excess body fatness in New Zealand children. Asia Pac J Clin Nutr.

[33] Shan XY, Xi B, Cheng H, Hou DQ, Wang Y, Mi J (2010). Prevalence and behavioral risk factors of overweight and obesity among children aged 2-18 in Beijing, China. Int J Pediatr Obes.

[34] Chiang PH, Wahlqvist ML, Lee MS, Huang LY, Chen HH, Huang ST (2011). Fast-food outlets and walkability in school neighbourhoods predict fatness in boys and height in girls: a Taiwanese population study. Public Health Nutr.

[35] Andegiorgish AK, Wang J, Zhang X, Liu X, Zhu H (2012). Prevalence of overweight, obesity, and associated risk factors among school children and adolescents in Tianjin, China. Eur J Pediatr.

[36] Xu H, Short SE, Liu T (2013). Dynamic relations between fast-food restaurant and body weight status: a longitudinal and multilevel analysis of Chinese adults. J Epidemiol Community Health.

[37] Hua J, Seto E, Li Y, Wang MC (2014). Development and evaluation of a food environment survey in three urban environments of Kunming, China. BMC Public Health.

[38] Zhang X, van der Lans I, Dagevos H (2012). Impacts of fast food and the food retail environment on overweight and obesity in China: a multilevel latent class cluster approach. Public Health Nutr.

[39] Xue H, Wu Y, Wang X, Wang Y (2016). Time trends in fast food consumption and its association with obesity among children in China. PLoS One.

[40] Ji CY (2005). Report on childhood obesity in China (1)--body mass index reference for screening overweight and obesity in Chinese school-age children. Biomed Environ Sci.

[41] Meng LH, Mi J, Cheng H, Hou DQ, Zhao XY, Ding XY (2008). Using waist circumference and waist-to-height ratio to access central obesity in children and adolescents. Chin J Evid Based Pediatr.

[42] Mi J, Wang TY, Meng LH, Zhu GJ, Han SM, Zhong Y (2010). Development of blood pressure reference standards for Chinese children and adolescents. Chin J Evid Based Pediatr.

[43] Xi B, Liang Y, Mi J (2013). Hypertension trends in Chinese children in the national surveys, 1993 to 2009. Int J Cardiol.

[44] Hsu YW, Johnson CA, Chou CP, Unger JB, Sun P, Xie B (2011). Correlates of overweight status in Chinese youth: an east-west paradox. Am J Health Behav.

[45] Adair LS, Popkin BM (2005). Are child eating patterns being transformed globally?. Obes Res.

